# Construction of a *Salmonella* Gallinarum ghost as a novel inactivated vaccine candidate and its protective efficacy against fowl typhoid in chickens

**DOI:** 10.1186/1297-9716-43-44

**Published:** 2012-05-23

**Authors:** Atul A Chaudhari, Chetan V Jawale, Sam Woong Kim, John Hwa Lee

**Affiliations:** 1 College of Veterinary Medicine, Chonbuk National University, Jeonju, 561-756, Republic of Korea

## Abstract

In order to develop a novel, safe and immunogenic fowl typhoid (FT) vaccine candidate, a *Salmonella* Gallinarum ghost with controlled expression of the bacteriophage PhiX174 lysis gene *E* was constructed using pMMP99 plasmid in this study. The formation of the *Salmonella* Gallinarum ghost with tunnel formation and loss of cytoplasmic contents was observed by scanning electron microscopy and transmission electron microscopy. No viable cells were detectable 24 h after the induction of gene *E* expression by an increase in temperature from 37 °C to 42 °C. The safety and protective efficacy of the *Salmonella* Gallinarum ghost vaccine was tested in chickens that were divided into four groups: group A (non-immunized control), group B (orally immunized), group C (subcutaneously immunized) and group D (intramuscularly immunized). The birds were immunized at day 7 of age. None of the immunized animals showed any adverse reactions such as abnormal behavior, mortality, or signs of FT such as anorexia, depression, or diarrhea. These birds were subsequently challenged with a virulent *Salmonella* Gallinarum strain at 3 weeks post-immunization (wpi). Significant protection against the virulent challenge was observed in all immunized groups based on mortality and post-mortem lesions compared to the non-immunized control group. In addition, immunization with the *Salmonella* Gallinarum ghosts induced significantly high systemic IgG response in all immunized groups. Among the groups, orally-vaccinated group B showed significantly higher levels of secreted IgA. A potent antigen-specific lymphocyte activation response along with significantly increased percentages of CD4^+^ and CD8^+^ T lymphocytes found in all immunized groups clearly indicate the induction of cellular immune responses. Overall, these findings suggest that the newly constructed *Salmonella* Gallinarum ghost appears to be a safe, highly immunogenic, and efficient non-living bacterial vaccine candidate that protects against FT.

## Introduction

The Gram-negative bacterium, *Salmonella enterica* serovar Gallinarum (*Salmonella* Gallinarum), is one of the major intracellular bacterial pathogens that causes fowl typhoid (FT), a septicemic disease of domestic birds, primarily chickens [1,2]. *Salmonella* Gallinarum infection is specifically limited to avian species and rarely causes food poisoning in humans [2]. FT is manifested by acute mortality, usually 60–70%, and severe inflammation including hepatitis, splenitis, typhlitis, and omphalitis, which results in significant economic losses to the poultry industry worldwide. The eradication of this disease is extremely problematic in countries where the ambient temperature necessitates the use of open-sided housing [3-5].

There have been several attempts to prevent FT by vaccination [5-9]. The rough mutant strain *Salmonella* Gallinarum 9R developed 50 years ago has been examined due to its protective efficacy against FT [9]. However, the use of live *Salmonella* Gallinarum 9R vaccine is limited to layer breeds older than 6-weeks and is associated with several disadvantages such as insufficient protection, low growth rate [6], and residual virulence that can cause hepatitis and splenic lesions in chicks [10]. Other trials with live attenuated *Salmonella* vaccines have shown successful induction of both mucosal and systemic immunity [11,12]. However, the major drawbacks with using these live vaccines are the safety of the animals and environmental contamination via fecal shedding. The alternative for these live vaccines could be the use of subunit or inactivated vaccines. The administration of a subunit vaccine along with an adjuvant has been shown to offer better protection than an experimental live vaccine [6]. However, common inactivation methods such as the use of formalin or heat for producing conventional inactivated vaccines often result in reduced or altered antigenic characteristics of the vaccines, and could be responsible for inducing impaired immune responses [13,14]. Therefore, a vaccine that can be safely administered to chickens (especially at a young age) to obtain desired immune responses and offer sufficient protection from FT is needed.

The bacterial ghost (BG) represents an innovative approach in non-living vaccine technology for producing safe and potent vaccines against a wide variety of infectious diseases [15-18]. Bacterial ghosts are empty cell envelopes that are produced by the controlled expression of the *phi*X17*4* lysis gene *E* in Gram-negative bacteria. Expression of the lysis gene *E* leads to the formation of trans-membrane tunnels through which the cytoplasm containing the bacterial genome [15,18] and plasmids is expelled due to high osmotic pressure inside the cell. The resulting bacterial ghosts are believed to retain all functional, morphological, and immunogenic determinants on the cell surface, and are able to induce cellular and humoral immune responses, resulting in effective immunoprotection [19]. Following ghost formation, even highly sensitive and fragile structures such as pili are preserved [20,21]. Since lysis tunnel formation is restricted to only a small part of the total cell surface and lysis by gene *E* does not chemically or physically disrupt bacterial surface structures, the resulting ghosts share the functional and antigenic determinants of the envelope with their living counterparts; thus, the ghosts can elicit prolonged and profound immunity [22-25]. In this study we constructed *Salmonella* Gallinarum ghosts using a plasmid containing the *phi*X17*4* lysis gene *E*. These *Salmonella* Gallinarum ghosts appear to be a safe vaccine candidate that significantly induces cellular and humoral immune responses, and confers significant protection against virulent *Salmonella* Gallinarum infection in chickens.

## Materials and methods

### Ghost plasmid construction and growth conditions

Bacterial strains and plasmids used in this study are listed in Table 1. Plasmid pHCE GAPDH ghost 37SDM carrying the parent ghost cassette was constructed as previously described [26]. PCR amplification of the ghost cassette was performed using pHCE GAPDH ghost 37SDM as a template and the primers ghost-F-XbaI (5′- TCTAGAGACCAGAACACCTTGCCGATC-3′) and ghost-R-XbaI (5′- TCTAGAACATTACATCACTCCTTCCG-3′). The amplified DNA segment was cloned into a T-easy vector (Promega, Madison, WI, USA), and designated as pMMP99. JOL394, a wild-type *Salmonella* Gallinarum (*Salmonella* Gallinarum) strain, was used for the construction of the ghost vaccine strain. The plasmid pMMP99 was used to transform JOL394 cells on LB agar containing 50 μg/mL of ampicillin; the resulting strain was denoted as JOL1277. All strains were preserved in LB broth with 20% glycerol and stored at −80 °C until use. Phosphate buffered saline (PBS, pH = 7.4) was used to resuspend the *Salmonella* Gallinarum ghost vaccine as well as the challenge strain.

**Table 1:** Bacterial strains and plasmids used in this study

**Strains/plasmid**	**Description**	**References**
***E. coli***		
Top 10	F-*mcr*A (*mrr-hsd*RMS-*mcr*BC) F80 *lac*Z M15 *lac*X74 *rec*A1 *ara* 139(*ara-leu*)7697 *gal*U *gal*K *rps*L (Str^r^) *end*A1 *nup*G	Invitrogen
***Salmonella*Gallinarum**		
JOL394	*Salmonella* Gallinarum, isolated from chicken	This study
JOL 1277	*Salmonella* Gallinarum JOL394 containing pMMP99	This study
**Plasmids**		
T-vector	Cloning vector; pUC*ori* Amp^R^	Promega
pMMP99	A derivative of T-easy carrying ghost cassette	This study

### Production of the *salmonella* gallinarum ghosts

JOL1277 cells harboring pMMP99 were inoculated into 20 mL of LB broth containing 50 μg/mL of ampicillin. The cultures were incubated at 37 °C with slow agitation until the optical density (OD) at 600 nm was 0.3–0.4. To induce gene *E*-mediated lysis, the temperature was increased to 42 °C. After increasing the temperature from 37 to 42 °C, lysis was monitored by performing viable cell counts at different time points (6, 12, 18 and 24 h). After completion of lysis, bacterial ghosts were harvested by centrifugation (4000 × *g* for 10 min), washed three times with sterile PBS, suspended in PBS, and stored at-20 °C.

### Scanning electron microscopy (SEM) and transmission electron microscopy (TEM)

The morphological features of the *Salmonella* Gallinarum ghosts (JOL1277) and a *Salmonella* Gallinarum wild-type strain (JOL394) as a control were examined by scanning electron microscopy (SEM) and transmission electron microscopy (TEM) as previously described with minor modifications [27,28]. Briefly, cell materials were harvested by centrifugation (4000 × *g* for 10 min) and fixed in 2.5% glutaraldehyde in PBS (pH 7.4). This was followed by post-fixation in 1% aqueous osmium tetroxide and then serial dehydration in acetone. Samples were critical point dried and coated with a gold-palladium alloy for subsequent observation by SEM (JSM-5200, JEOL, Japan). To prepare negatively-stained TEM specimens, a drop of the *Salmonella* Gallinarum ghost suspension was placed on carbon-coated copper grids. The grids were allowed to stand for 2 min to permit films to form on the grids, the extra solution was removed using blotting paper, and the grids were dried. TEM was performed with a HITACHI-JP/H7600 microscope (Hitachi Science Systems, Japan) operated at an accelerating voltage of 100 kV.

### Evaluating the safety dose of the *salmonella* gallinarum ghost vaccine candidate in chickens

All animal experiments described in this study were conducted with approval (CBU 2011–0017) from the Chonbuk National University Animal Ethics Committee in accordance with the guidelines of the Korean Council on Animal Care. One-day old female Brown Nick chickens were used for all the experiments, and provided with water and antibiotic-free feed *ad libitum*. To determine the safety and appropriate dose for inoculation of a ghost vaccine, a preliminary experiment was carried out in chickens. The chickens divided into three groups (*n* = 15 per group), were inoculated via oral route using 10^7^, 10^8^ and 10^9^ bacterial ghosts at day 7 of age. The control group was inoculated orally with PBS. The general condition of the birds was evaluated daily until 3 weeks post-immunization (wpi). Mortality and the development of clinical symptoms of FT such as anorexia, depression, and greenish-tinted diarrhea were monitored. Loss of appetite, feed residue, and low responsiveness to external stimuli were monitored to identify anorexia and depression. The birds were subsequently challenged at 21 days post-immunization with 100 μL of a suspension containing 1 × 10^6^ CFU of a wild-type *Salmonella* Gallinarum JOL394 strain and assessed for mortality. Before the actual vaccination-challenge experiment, a small experiment was carried out to monitor the adverse effects of the intramuscular and subcutaneous inoculation with *Salmonella* Gallinarum ghost vaccine in chickens. The birds (*n* = 10/route) were inoculated with PBS containing 10^8^*Salmonella* Gallinarum ghosts via the intramuscular and subcutaneous route. The control birds (*n* = 10/route) received only PBS. None of the birds exhibited any adverse inflammatory reactions when inoculated with *Salmonella* Gallinarum ghost vaccine or PBS by intramuscular and subcutaneous routes.

### *Salmonella* gallinarum ghost vaccine protection against bacterial challenge

Based on the previous safety dose evaluation data, *Salmonella* Gallinarum ghosts were inoculated at 10^10^ inoculation dose by oral route and 10^8^*Salmonella* Gallinarum ghosts (100 times less than the oral dose) for intramuscular or subcutaneous inoculation. All groups of chickens were immunized with the bacterial ghosts at the 7^th^ day of age. In group B, the birds were orally inoculated with the *Salmonella* Gallinarum ghosts at a concentration of 1 × 10^10^ cells/0.1 mL/chicken. In group C, the chickens subcutaneously received the ghosts in 0.1 mL of a suspension containing 1 × 10^8^ cells/chicken. Group D birds were injected intramuscularly with 0.1 mL of the *Salmonella* Gallinarum ghost suspension containing 1 × 10^8^ cells/chicken. The control group A was kept as non-immunized control. Post immunization, the birds were monitored for mortality and any detrimental effects induced by *Salmonella* Gallinarum ghost vaccine as described above. The birds in all groups were challenged with 100 μL of a suspension containing 1 × 10^6^ CFU of a wild-type *Salmonella* Gallinarum JOL394 strain on day 21 post-immunization. The mortality rate was assessed daily for 14 days after the challenge. All birds were then sacrificed and examined for macroscopic lesions. Lesion scores were determined and recorded using a system similar to the one previously described [29-31] with modifications. Each gross hepatic and splenic lesion was assigned a score of 0, 1, 2, or 3. Hepatic and splenic lesion scores were considered moderate or severe depending on organ enlargement and the presence of white necrotic foci. A score of 0 indicated no lesions whereas a score of 1 was assigned to indicate hepatomegaly or splenomegaly. The presence of over 10 white necrotic foci on the liver and spleen was scored as 2, and the presence of more than 10 white foci along with the liver and spleen enlargement was scored as 3. The lesion score for dead birds in all groups was 3. A mean lesion score per group was determined, and differences among the groups were statistically analyzed. Upon post-mortem examination, the presence of the challenge strain in internal organs of birds with FT lesions was investigated. For bacterial recovery, a sample of the liver and spleen was directly inoculated on BGA and incubated overnight at 37 °C. Thereafter, the organs were minced and suspended in buffered peptone water (Becton, Dickinson and Company), and the homogenate was incubated overnight at 37 °C with continuous shaking, followed by culture in Rappaport- Vassiliadis R10 broth (Becton, Dickinson and Company) for 48 h at 42 °C. A loop of the enrichment broth was streaked onto BGA and the *Salmonella*- type colonies were examined after incubation at 37 °C for 24 h.

### Collection of plasma and samples for measuring intestinal secretory IgA (sIgA)

Plasma and intestinal washes were collected from five birds in each group to evaluate the immune responses at each week post-immunization. The plasma samples were obtained by centrifugation of the peripheral blood. The intestinal wash samples were collected as previously described with some modifications [32]. Briefly, the birds were orally administered a lavage solution [(6 mM polyethylene glycol (PEG; 3350 molecular weight), 40 mM Na_2_SO_4_, 20 mM NaHCO_3_, 10 mM KCl; and 25 mM NaCl, pH = 7.6)] at 5 mL/kg of body weight. The birds were individually placed in clean empty buckets and monitored for 30 min until they began to excrete feces. At this time, a sterile solution of 5% pilocarpine (Sigma, St. Louis, MO, USA) in water was injected intramuscularly at 50 mg/kg body weight. Administration of pilocarpine was followed by immediate and intermittent excretion of intestinal fluid for 30 min. After collection, the intestinal fluid was vortexed and centrifuged at 700 × *g* for 10 min. One mL of the supernatant was saved and added to 10 μL of a 100 mM phenylmethanesulfonyl fluoride (PMSF; Sigma,) solution, 10 μL of 1% (w/v) sodium azide, and 10 μL of 5% (w/v) bovine serum albumin. The samples were stored at −20 °C until use.

### Humoral immune response evaluation

An indirect enzyme-linked immunosorbant assay (ELISA) was performed with an outer membrane protein (OMP) extracted from the JOL394 *Salmonella* Gallinarum as described before [30]. The presence of plasma IgG and intestinal sIgA against the OMP was identified using a chicken IgG and IgA ELISA quantitation kit (Bethyl Laboratories, Montgomery, TX, USA). The wells of a Microlon® ELISA plate (Greiner Bio-One GmbH, Frickenhausen, Germany) were coated with 100 μL of OMP at a concentration of 0.2 mg/mL. Wells were incubated with a 1:250 dilution of plasma for 1 h, followed by incubation with a 1:100 000 dilution of horseradish peroxidase (HRP)-conjugated goat anti-chicken IgG (Bethyl Laboratories, Montgomery, TX, USA) for 1 h. The bound HRP activity was measured using *o*-phenylmethylsulfonyl fluoride (Sigma-Aldrich). The optical density at 492 nm was measured with an ELISA reader after the reaction was stopped with 50 μL of 3 M sulfuric acid. The sIgA concentrations were quantified using procedures similar to the ones that were used to measure plasma IgG levels (except that the intestinal wash samples were diluted 1:100).

### Cellular immune response evaluation

#### Antigen-specific cell activation of peripheral blood mononuclear cells

A lymphocyte activation assay was carried out as previously described to evaluate cell-mediated immunity in the immunized groups [33]. Soluble antigen was prepared from the *Salmonella* Gallinarum wild-type strain JOL394 as previously described [31]. Briefly, the bacterial cell suspension was sonicated for 2 min, and centrifuged at 5000 × *g* for 60 min at 4 °C. Supernatant containing the sonicated bacterial cell protein suspension (sbcp) was used as the soluble antigen. Peripheral blood mononuclear cells (PBMC) were separated from five chickens randomly selected from each group using a gentle swirl technique [34] on day 21 post-immunization. Trypan blue dye exclusion testing was done to determine cell viability. A 100 μL suspension of viable mononuclear cells containing 1 × 10^5^ cells/mL added to RPMI-1640 medium supplemented with 10% fetal calf serum, 2 mM L-glutamine, 50 U/mL penicillin, 50 μg/mL streptomycin, and 2 μg/mL fungizone. The suspension was incubated in triplicate in the wells of 96-well tissue culture plates with 50 μL of medium alone (negative control) or medium containing 4 μg/mL of sbcp at 40 °C in a humidified 5% CO_2_ atmosphere for 72 h. The 10 μg/mL Concanavalin A (ConA, Sigma, St. Louis, MO, USA) stimulated lymphocyte cells were kept as a positive control and activity of the stimulated cell suspension was measured. Activation of stimulated lymphocytes was measured using adenosine triphosphate (ATP) bioluminescence as a cell viability marker with a ViaLight® Plus Kit (Lonza, Rockland, ME, USA), which estimated mitochondrial activity [35]. The emitted light intensity was measured using a TriStarLB941 luminometer (Berthold Technologies GmbH and Co., Bad Wildbad, Germany) with an integrated program of 1 s duration. The values for each group are expressed as relative luminescence units (RLU).

#### Flow cytometry analysis of CD3^+^, CD4^+^ and CD8^+^ subsets

T cell markers such as CD3^+^, CD4^+^, and CD8^+^ were examined by collecting peripheral blood mononuclear cells (PBMC) from all the groups on day 7 post-immunization [36]. The cell suspensions were prepared at a concentration of 1 × 10^6^ cells/mL in cold PBS. The cells were washed three times in PBS and then incubated with 0.1 mL of appropriately diluted fluorescein isothiocyanate (FITC)-labeled anti-CD3, biotin (BIOT)-labeled anti-CD4 and phycoerythrin (PE)-labeled anti-CD8a (SouthernBiotech, Birmingham, AL, USA) monoclonal antibodies in the dark at 4 °C for 30 min. After washing three times with cold PBS, the cells were incubated with 0.1 mL of appropriately diluted allophycocyanin (APC)-labeled streptavidin (SouthernBiotech, Birmingham, USA) monoclonal antibody in the dark at 4 °C for 30 min. After incubation, all samples were washed three times with cold PBS, resuspended in 0.5 mL of PBS, and analyzed with a flow cytometer (BD Biosciences, NJ, USA). FACS analysis of 10 000 events was performed using CellQuest software (BD Biosciences).

### Statistical analysis

All data are expressed as the mean ± standard deviation (SD) unless otherwise specified. Analyses were performed with SPSS version 16.0 software (SPSS, Chicago, IL, USA). A non-parametric chi-square test was used to analyze significant differences in mortality and gross lesion scores. A one way ANOVA with post hoc Bonferroni adjustments was used to analyze statistical differences in immune responses between the immunized groups and unimmunized control group. Differences were considered to be statistically significant when the *P-*values were ≤ 0.05 or 0.01.

## Results

### Construction of the ghost cassette and lysis of *salmonella* gallinarum bacterial ghosts

The ghost cassette contained the cI P_R_ promoter from bacteriophage lambda and *E* gene from phiX174 fused at transcriptional and translational levels. A new ghost cassette designated as ghost SDM37 was constructed as described in a previous study [26]. The SDM37 ghost system was prepared by introducing a mutation in an operator region encoding the *R* gene. The point-mutated site in ghost SDM37 was located at OR2 which consisted of 17 nucleotides (nts). Among the 17 nts, the ninth nucleotide, C, was replaced with T in ghost SDM37. Because of this, the strain carrying the SDM37 ghost system could grow at temperatures lower than 37 °C but were unable to grow at temperatures higher than 37 °C due to the induction of lysis.

A plasmid (pMMP99) containing the ghost cassette was used to transform a wild-type *Salmonella* Gallinarum strain, JOL394. Production of *Salmonella* Gallinarum ghosts in a 25 mL liquid culture of randomly selected clones was accomplished by increasing the temperature up to 42 °C to activate gene *E*-mediated lysis. The OD of the transformed *Salmonella* Gallinarum culture decreased during 6 h after induction of gene *E* expression due to cell lysis and remained constant for the next 24 h until the bacterial ghosts were harvested (Table 2). Cell viability also decreased after lysis induction until the time of ghost harvesting (Table 2). The concentration of viable bacteria, 6 h after lysis induction, decreased 10^5^-fold orders of magnitude (Table 2). Lysis was completed 24 h after the initiation at which time no viable cells were detected.

**Table 2:** **Viable cell count after induction of gene*E*mediated lysis**

**Incubation hours**^**a**^	**OD**_**600**_	**CFU**^**b**^
0	0.320	2.5 × 10^9^
6	0.188	1.8 × 10^4^
12	0.178	4 × 10^3^
18	0.150	1 × 10^3^
24	0.167	0

^a^ The cultures were collected at different time intervals such as 0, 6, 12, 18 and 24 h after the induction of lysis.

^b^ The viable cell count was measured as colony forming units (CFU)/mL.

**Figure 1:**
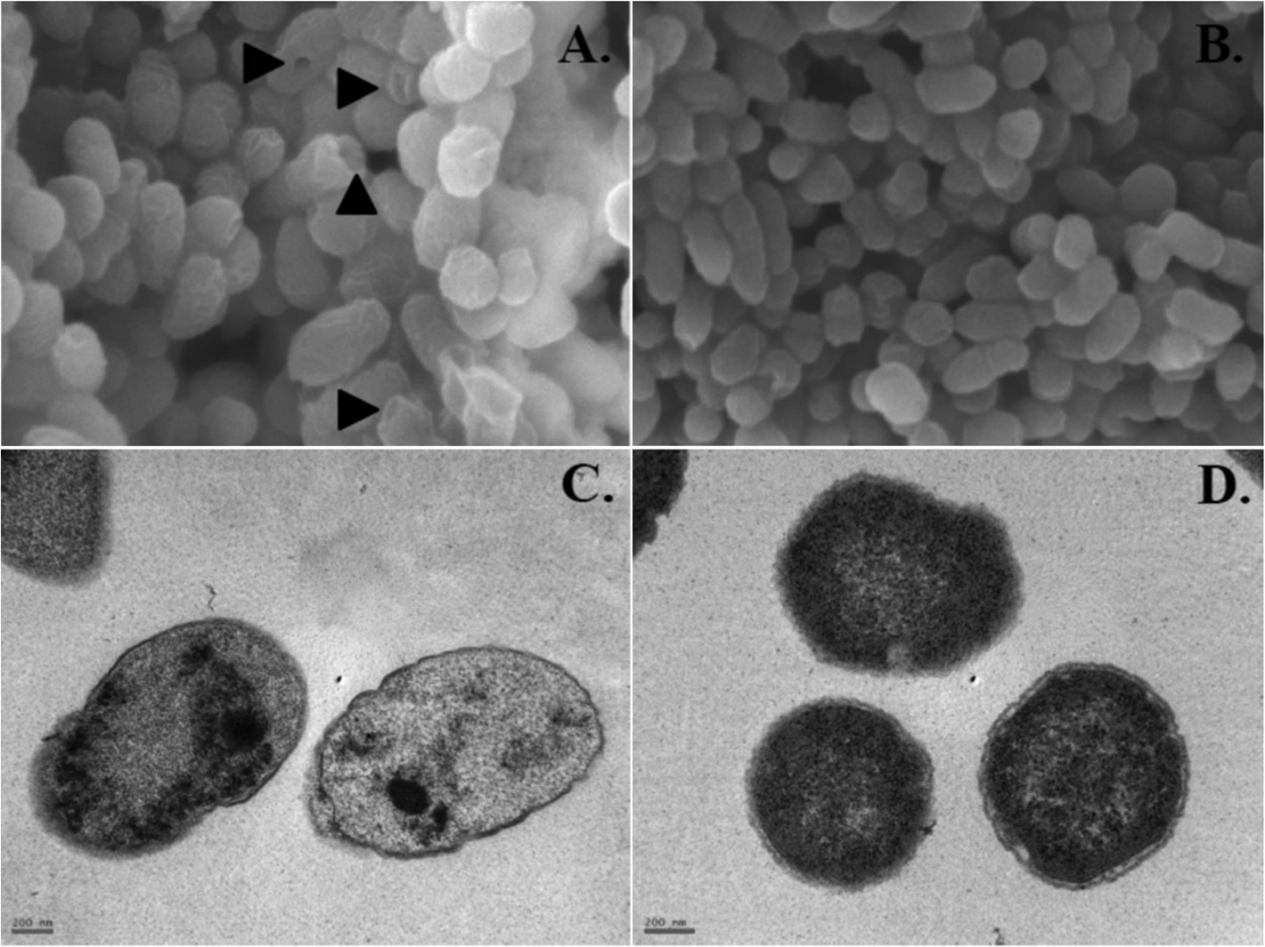
**Evaluation of*Salmonella*Gallinarum ghosts (JOL1277) and*Salmonella*Gallinarum (JOL394) by SEM (A and B) and TEM (C and D).A**) *Salmonella* Gallinarum ghosts. Arrow shows the trans-membrane lysis tunnel. **B**) Naive *Salmonella* Gallinarum examined by SEM. **C**) Loss of cytoplasmic material from *Salmonella* Gallinarum ghosts. **D**) Naive *Salmonella* Gallinarum examined by TEM.

Formation of the *Salmonella* Gallinarum ghosts was examined by SEM and TEM. Electron microscopic analysis revealed the formation of lysis pores in the *Salmonella* Gallinarum ghosts (indicated by arrowheads in Figure 1a). Pores resulting from gene *E* tunnel formation could be seen either in the pole region or central division region (Figure 1a). TEM examination showed that the *Salmonella* Gallinarum ghost cells were empty due to the loss of cytoplasmic material and had collapsed cell envelopes compared to the wild-type cells (Figure 1b).

### Safety of the ghost vaccine candidate in chickens

Seven-day-old chickens were inoculated with the *Salmonella* Gallinarum ghost strain. To evaluate the safety of the vaccine, parameters including clinical symptoms and mortality were measured post-immunization. No vaccination associated mortality was observed in any of the immunized groups. Furthermore, the birds remained healthy without any symptoms of anorexia, diarrhea, or depression for 3 weeks after inoculation with the ghost strain.

### Humoral immune responses

**Figure 2:**
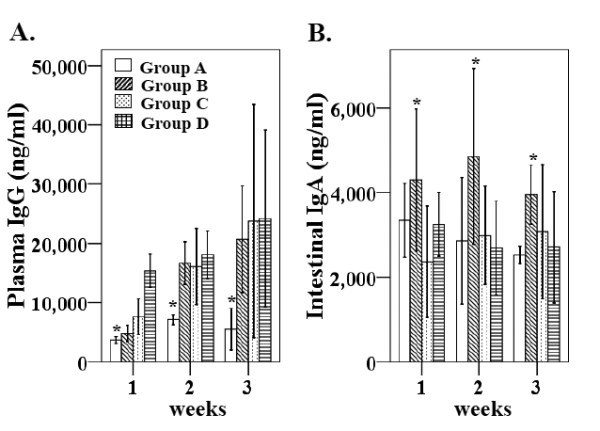
**The plasma IgG and intestinal sIgA levels in chickens against the outer membrane protein (OMP).A**) Plasma IgG levels against specific antigens were determined with the chicken IgG quantitative ELISA. **B**) The sIgA levels in intestinal wash fluids against OMP antigen were quantified using the chicken IgA quantitative ELISA. Antibody levels were expressed as mean ± S.D. values for each week post-immunization. The values were considered statistically significant, if *p* values were ≤ 0.05 (*p ≤ 0.05). Group A, non-immunized control; group B, orally immunized; group C, immunized subcutaneously; group D, intramuscularly immunized.

Humoral immune responses specific for the OMP antigen extracted from wild-type *Salmonella* Gallinarum were evaluated by indirect ELISA. Systemic IgG and mucosal sIgA levels were determined at every week post-immunization by measuring specific IgG levels in plasma and IgA concentrations in intestinal wash fluid. The immunized birds in group B (orally immunized), group C (subcutaneously immunized), and group D (intramuscularly immunized) had significantly higher plasma IgG levels than the group A control birds (Figure 2a) every week post-immunization (wpi). The increases in IgG levels at 3 wpi in the immunized groups were highly significant compared to the non-immunized group (Figure 2a). Sequential monitoring of intestinal sIgA antibodies showed significantly increased levels in the orally immunized group B compared to the control group (Figure 2b). There was no significant increase in intestinal sIgA levels in groups C or D (Figure 2b).

### Cellular immune responses

#### Lymphocyte activation assay

**Figure 3:**
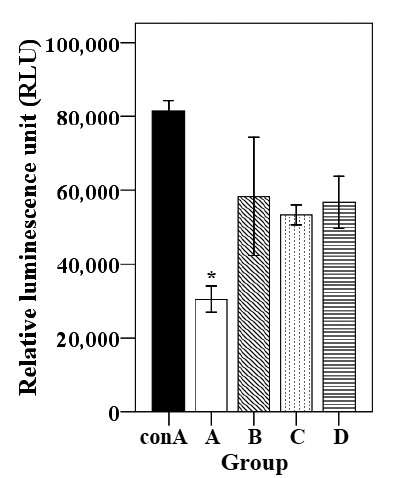
**The lymphocyte stimulation responses determined at 3-week-postimmunization against the sbcp Antigen.** The stimulation index of lymphocyte sample from the chickens was determined by the peripheral lymphocyte activation assay. The values were considered statistically significant, if *p* values were ≤ 0.05 (*p ≤ 0.05). Group A, non-immunized control; group B, orally immunized; group C, immunized subcutaneously; group D, intramuscularly immunized.

In order to evaluate the cell-mediated immune responses, lymphocyte activation after stimulation with soluble antigen was measured by analyzing PBMC isolated from immunized and non-immunized birds at 3 wpi. The immunized birds showed significant activation responses compared to control group A (Figure 3). The immunized group RLU values differ significantly compared to those of the control group, suggesting a dramatic increase of lymphocyte activation after antigen stimulation.

#### Flow cytometry analysis of CD4^+^ and CD8^+^ T cells

**Figure 4:**
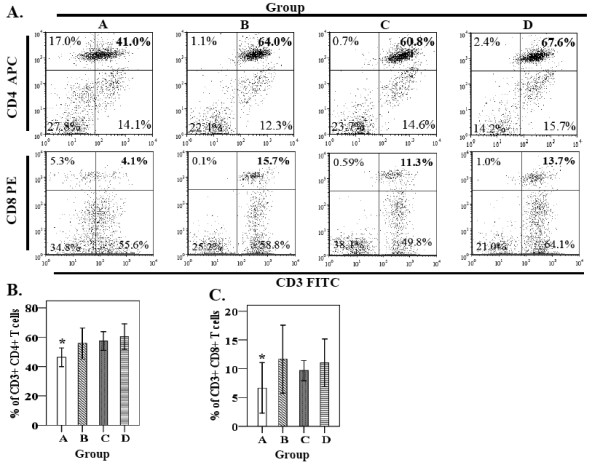
**Flow cytometric analysis for CD3**^**+**^**CD4**^**+**^**and CD3**^**+**^**CD8**^**+**^**T-lymphocyte populations.A**) Flow cytometry scatter dot plots for CD3^+^, CD4^+^, CD8^+^ T-cell populations. The plots represent events for one representative chicken from each group. The gating and the quadrants are set according to the standard procedures of the BD-Biosciences flow cytometer **B**) Bar-graphs represent CD3^+^ CD4^+^ T-lymphocyte populations in immunized and non-immunized chickens **C**) Bar-graphs represent CD3^+^ CD8^+^ T-lymphocyte populationin immunized and non immunized chickens. Values are shown as Mean ± S.D of 5 chickens per group and were considered statistically significant, if *p* values were ≤ 0.05 (*p ≤ 0.05). Group A, non- immunized control; group B, orally immunized; group C, immunized subcutaneously; group D, intramuscularly immunized.

Changes in T cell populations after immunization correlated with comparable changes in serum antibody levels. Therefore, we measured the CD4^+^ (T helper cells) and CD8^+^ (cytotoxic T cells) T cell subpopulations among PBMC from the immunization groups by flow cytometry. The relative numbers of both CD4^+^ and CD8^+^ lymphocytes significantly increased in all the immunized groups (Figure 4). The immunized group had statistically (*p* < 0.05) higher numbers of CD3^+^ CD4^+^, and CD3^+^ CD8^+^ T cells than the non-immunized control group (Figure 4a and b). On day 7 post-immunization, the numbers of CD3^+^ CD4^+^ and CD3^+^ CD8^+^ T cells among the PBMC populations of all immunized chickens were approximately 1.5- and 2.5-fold greater than the non-immunized control group, respectively (Figure 4).

### Protective efficacy of bacterial ghosts against virulent challenge in chickens

In the preliminary experiment for determination of safety dose for inoculation, it was observed that the birds immunized with 10^7^, 10^8^ and 10^9^ showed 46.6, 33.5 and 26% mortality against the virulent challenge, respectively (Table 3). Based on this, the birds were immunized with 10^10^ and 10^8^ bacterial ghosts via oral and intramuscular/subcutaneous routes, respectively and were subsequently inoculated via oral route with the challenge strain at 3 wpi to investigate the protective efficacy of the *Salmonella* Gallinarum ghosts against virulent challenge. The birds were subsequently observed for mortality for 14 days post-challenge. Immunized groups B, C, and D showed significant protection against the challenge compared to the non-immunized group A (Table 4). The immunized groups exhibited significantly lower mortality than the non-immunized control group A, which showed 73% mortality (Table 4). The percent mortality rates for groups B, C, and D were 14.0%, 6.6%, and 6.6%, respectively. At day 14 post-challenge, in each group the surviving chickens were euthanized for postmortem examination to evaluate the protective efficacy of the ghost vaccine. The presence of hepatic and splenic enlargement with white necrotic spots was scored from 0 to 3 as described in “Materials and Methods”. The mean organ lesion scores of the immunized groups were compared to those of the non-immunized control group A. All the immunized groups (B, C, and D) had significantly lower lesion scores than those of the control group A (Table 4). The challenge strain was not isolated from the internal organs of any of the chicks of groups at day 14 post challenge.

**Table 3:** **Safety dose evaluation of the*Salmonella*Gallinarum ghost vaccine candidate in chickens**

**Group**	**Dose**^**a**^	**Mortality after Challenge**^**b**^**(%)**
Vaccinated	10^7^	7/15 (46.6)
	10^8^	5/15 (33.5)
	10^9^	4/15 (26.6)
Control	-	11/15 (73.6)

^a^ The birds were inoculated with 1 × 10^7^, 1 × 10^8^ and 1 × 10^9^ of the *Salmonella* Gallinarum ghost vaccine.

^b^ Number of dead birds upon challenge.

**Table 4:** Mortality and gross lesion in the chickens post challenge

**Group**^**a**^	**Challenge**^**b**^				
	**Gross lesion**^**d**^			**Bacterial recovery**	
	**Mortality (%)**	**Liver**	**Spleen**	**Liver**	**Spleen**
				**Positive No.**	**Positive No.**
A	11/15 (73)^c^	2. 2.16 ± 1.46^e**^	1.70 ± 1.46^**^	0/5^f^	0/5
B	2/15 (14)	1.06 ± 0.96	0.66 ± 1.12	0/5	0/5
C	1/15 (6.6)	0.60 ± 0.98	0.53 ± 0.83	0/5	0/5
D	1/15 (6.6)	0.53 ± 0.89	0.53 ± 0.93	0/5	0/5

^a^ Immunization was performed at day 7 of age with the *Salmonella* Gallinarum ghost strain and the groups were designated as group A (non vaccinated), group B (orally immunized), group C (immunized subcutaneously) and group D (immunized intramuscularly). Fifteen birds were allocated per group.

^b^ Challenge was performed with a wild type *Salmonella* Gallinarum strain using 1 × 10^6^ CFU after 21 days post vaccination.

^c^ Number of dead birds upon challenge.

^d^ Gross lesion was observed at day14 post challenge.

^e^ Group lesion score (Mean ± SD).

^f^ Number of positive samples after enrichment culture. All values were considered to be significant if *p* ≤ 0.05 or 0.01.* *p* < 0.05, **p < 0.01 vs. non vaccinated group A.

## Discussion

To vaccinate against FT, it is necessary that the vaccine contains either live *Salmonella* vaccine strains of reduced virulence, or inactivated organisms. In the present study we constructed a *Salmonella* Gallinarum ghost vaccine and evaluated its protective efficacy in chickens. Bacterial ghost (BG) represents a relatively novel concept for genetically engineered vaccine technology. However, this new ghost system has been rarely used in poultry. BG are devoid of all cytoplasmic content but preserve their natural outer membranes with highly immunogenic lipopolysaccharide structures. As reported in this paper, we succeeded in producing *Salmonella* Gallinarum ghosts as a result of lysis gene *E* expression followed by formation of transmembrane tunnel structures through which the cytoplasmic contents were expelled, leaving behind the empty cell envelopes (Figure 1). Culture solutions of *Salmonella* Gallinarum ghosts produced by this method did not contain any viable cells 24 h after lysis induction and no morphological changes were observed by SEM or TEM. The onset of lysis was observed 6 h after the temperature was increased to 42 °C with decreases in both OD_600_ and cell viability. Generally, strains carrying a conventional ghost cassette system are able to survive at 28 °C [37,38] whereas the ghost strain used in the present study was grown at 37 °C, which is the optimum temperature for normal growth of *Salmonella*[30], since sub-optimal growth temperatures reduce the rate of division and thus slowdown the production process of bacterial ghosts. Previous studies have indicated that growth of *Salmonella* at lower than 30 °C affected the lipopolysaccharide O-antigen size and distribution [39] and reduced the expression of certain fimbriae [40]. These changes in the surface structures may account for the alteration in antigenicity of *Salmonella* Gallinarum ghost. A ghost strain, which was initially grown at 37 °C and then exposed to 42 °C to promote the lysis, resulted in efficient induction of immune responses [16]. Based on this, the present study assumed that the *Salmonella* Gallinarum cells when grown under normal optimal temperatures required for *Salmonella* growth before the actual lysis induction could maintain their natural antigenic structure efficiently to trigger the desired immune responses.

It has been previously demonstrated that immunization with BG vaccines offer efficient protection against highly virulent strains in animal models [16,41]. Therefore, we further evaluated the safety and protective efficacy of the *Salmonella* Gallinarum ghosts as a vaccine candidate against infection with a wild-type strain in chickens immunized with *Salmonella* Gallinarum ghosts via either oral, intramuscular, or subcutaneous routes. None of the immunized groups showed any adverse reactions after vaccination which suggests that the ghost vaccine used in the present study could be safely administered in chickens without any detrimental effects. Immunization with the *Salmonella* Gallinarum ghosts offered significant protection against the virulent challenge based on mortality and the development of lesions (Table 4). Animals in all the immunized groups had significantly lower gross lesion scores compared to the non-immunized control group. Although no significant differences were observed among the immunized groups, the mortality rates and gross lesion scores for all the immunized groups differ significantly compared to those of the non-immunized group. The present study results are promising and may indicate that the newly constructed *Salmonella* Gallinarum ghost vaccine could be an effective measure to prevent FT. However, the advantages of the presently constructed *Salmonella* Gallinarum ghost vaccine over the previously reported live [10] or subunit vaccines [6] should be validated. This could be achieved via comparative evaluation studies between the *Salmonella* Gallinarum ghost vaccine and other live/inactivated vaccines.

*Salmonella* Gallinarum antigen-specific humoral and cellular immune responses induced by vaccination have been correlated with the protective efficacy of live vaccines against FT in chickens [33,42]. Unlike other inactivated vaccines, BG retain all relevant antigens found on live bacteria which can be recognized and engulfed by dendritic cells and macrophages in immunized animals, thereby stimulating humoral and cell-mediated immune responses [41]. Therefore, our study further investigated the humoral and cellular immune responses in chickens after immunization with the *Salmonella* Gallinarum ghosts. Our data indicate that immunization with the *Salmonella* Gallinarum ghosts efficiently induced systemic IgG responses in all immunized groups whereas mucosal sIgA levels were significantly elevated in group B (Figure 2a and b). The *Salmonella* Gallinarum ghost vaccine induced significantly high plasma IgG levels in chickens and the antibody levels continually increased until 3 wpi, indicating a strong systemic immune response in the chickens after immunization. Mucosal sIgA levels were significantly elevated in the orally immunized group. The stronger sIgA response was likely to be related to the fact that the oral route of immunization induces significantly higher mucosal secretory IgA levels compared to the other routes [43]. Thus, our data suggest that the oral immunization with the *Salmonella* Gallinarum ghosts had an additional advantage of inducing sIgA levels which serve as a first line of defense against enteric pathogens [43] and might play an important role in early elimination of *Salmonella* Gallinarum infections. Moreover, the role of sIgA or mucosal immunity in *Salmonella* Gallinarum clearance and systemic FT control has not been fully elucidated [42]. The present experimental data showing sIgA induction following oral immunization with 10^10^*Salmonella* Gallinarum ghosts may offer useful information about the induction of intestinal sIgA antibodies although its role in protection against the *Salmonella* Gallinarum clearance in the chicken is a matter of interest. Presently, our study indicates that immunization with an inactivated *Salmonella* Gallinarum ghost vaccine via oral route is capable of inducing mucosal immunity.

Cell-mediated immune responses play a central role in protecting against intracellular bacterial pathogens [16,33,44]. *Salmonella* Gallinarum is an intracellular pathogen, and has been reported to invade the mononuclear phagocyte system and survive in chicken macrophages [30]. Therefore, immunization against *Salmonella* Gallinarum infection requires a vaccine that can efficiently prevent the disease by inducing immune responses at the cellular level. The correlation between cellular immunity and high survival rates in chickens after a virulent *Salmonella* Gallinarum challenge has been reported, and measurements of cell-mediated immune responses is an important index of protection [30,42]. To evaluate the cellular immune responses, the present study performed an antigen-specific lymphocyte activation assay and, CD3^+^CD4^+^ and CD3^+^CD8^+^ T cell populations among the PBMC of the immunized and non-immunized groups. Our data showed that all immunized groups showed significant lymphocyte activation responses upon antigen stimulation compared to the non-immunized groups. These lymphocyte activation responses clearly suggest that the lymphocytes (either B or T cells) from the immunized birds were activated upon antigen stimulation, possibly due to circulating memory cells in the peripheral blood of the immunized birds. These findings may indicate that the *Salmonella* Gallinarum ghost vaccine could be as efficient as the other vaccines for activation of the lymphocytes.

In addition, we investigated dynamic changes in CD4^+^ and CD8^+^ T-lymphocyte populations in the immunized and non-immunized chickens. Analysis of the T cell populations in the respective vaccination groups revealed significant changes in T cell population profiles in all vaccinated groups compared to the non-immunized control group A (Figure 4). Our data show that vaccination with *Salmonella* Gallinarum ghosts induced a relative increase in the percentages of CD3^+^CD4^+^ and CD3^+^CD8+ T lymphocytes in the chickens. CD4^+^ T cells represent a major T cell population and are well known as T helper cells mainly associated with the Th1 and Th2 immune responses via the production of Th1 cytokines and increased antibody secretion, respectively [45]. CD8+ T cells, also known as cytotoxic T cells, play an important role in immune protection against intracellular bacterial parasites such as *Listeria monocytogenesMycobacterium tuberculosis,* and *Salmonella enterica*[45-47]. Thus, enhanced populations of these T cells in the immunized host are involved in protecting against intracellular pathogens. Increased CD4^+^ and CD8^+^ T cell populations in the chickens immunized with a live *Salmonella* Gallinarum mutant have been shown to correlate with protection against FT [36]. The data presented in this paper demonstrate that all immunized groups had CD4^+^ and CD8^+^ T cell populations and the percentages were significantly higher than those of the control group (Figure 4), indicating a strong cell-mediated immune response. The immune response observed in our study was similar to that induced in BG-vaccinated animals [14,37], and involved the generation of CD4^+^ and CD8^+^ T cells. Taken together, our data demonstrate that vaccination with the *Salmonella* Gallinarum ghosts induced a significant cellular immune response characterized by effective antigen-specific lymphocyte activation with enhanced production of T helper (CD4^+^) and cytotoxic (CD8^+^) T lymphocytes. However, it would be interesting to know whether there was an increase in the actual lymphocyte populations in the immunized groups in response to immunization and the relative increase in the percentages of the CD4^+^ and CD8^+^ T cells should be recalculated back to the real numbers to know if the changes were due to increases in these lymphocyte subpopulations.

In conclusion, the present study demonstrates that the vaccination of chickens with the *Salmonella* Gallinarum ghosts is a safe approach for preventing FT without inducing adverse clinical symptoms. Immunization with the *Salmonella* Gallinarum ghost not only led to significant immune responses but also provided efficient protection against experimentally-induced systemic FT. Taken together, these findings suggest that the newly constructed *Salmonella* Gallinarum ghost appears to be a promising vaccine candidate and can be used as a safe and highly immunogenic vaccine against FT although further safety and efficacy trials on the field are needed.

## Competing interests

The authors declare that they have no competing interests.

## Authors’ contributions

Conception and design of experiments: AC and JL. Conduction of experiments: AC, CJ and SK. Ghost cassette construction: SK. Animal experimentation and sample collection: AC and CJ. Manuscript draft preparation and data analysis: AC, JL and SK. Study supervision: JL. All authors have read and approved the final manuscript.
